# Tetrocarcin Q, a New Spirotetronate with a Unique Glycosyl Group from a Marine-Derived Actinomycete *Micromonospora carbonacea* LS276

**DOI:** 10.3390/md16020074

**Published:** 2018-02-24

**Authors:** Ting Gong, Xin Zhen, Xing-Lun Li, Jing-Jing Chen, Tian-Jiao Chen, Jin-Ling Yang, Ping Zhu

**Affiliations:** State Key Laboratory of Bioactive Substance and Function of Natural Medicines; Key Laboratory of Biosynthesis of Natural Products of National Health and Family Planning Commission, Institute of Materia Medica, Chinese Academy of Medical Sciences and Peking Union Medical College, 1 Xian Nong Tan Street, Beijing 100050, China; gongting@imm.ac.cn (T.G.); zhenxin@imb.pumc.edu.cn (X.Z.); lixinglun@imm.ac.cn (X.-L.L.); chenjingjing@imm.ac.cn (J.-J.C.); chentianjiao@imm.ac.cn (T.-J.C.); yangjl@imm.ac.cn (J.-L.Y.)

**Keywords:** marine-derived actinomycete, *Micromonospora*, spirotetronate glycoside, antibiotic, antibacterial activity

## Abstract

A new spirotetronate glycoside tetrocarcin Q (**1**) and six known analogues tetrocarcin A (**2**), AC6H (**3**), tetrocarcin N (**4**), tetrocarcin H (**5**), arisostatin A (**6**), and tetrocarcin F1 (**7**) were isolated from the fermentation broth of the marine-derived actinomycete *Micromonospora carbonacea* LS276. Their chemical structures were established on the basis of 1D- and 2D-NMR spectroscopy, as well as HR-ESI-MS analysis. The absolute configurations of their stereogenic carbons were determined by circular dichroism (CD) analysis. Compound **1** possesses 2-deoxy-allose, which is a unique sugar type at the C-9 position. This type has not been found in the previously reported spirotetronate glycosides. Compound **1** displayed moderate antibacterial activity against *Bacillus subitlis* ATCC 63501 with minimum inhibitory concentration (MIC) value of 12.5 μM.

## 1. Introduction

The spirotetronate family displays complicated chemical structures, potent bioactivities, and significant pharmacological potential [1]. This family features an unusual macrolide that contains a characteristic tetronic acid (spiro-linked to a cyclohexene ring) conjugated with a *trans*-decalin system. The structure is also linked with two sugar side chains, one of which is d-tetronitrose (NS), while the other comprises l-digitoxoses (DG) and l-amicetoses (AM) [2,3,4]. In terms of biological profile, the spirotetronate exhibits broad biological activities, including antibacterial, antitumor, antiviral, and antimalarial effects [5,6,7,8,9,10]. A representative of this group is the tetrocarcins, including tetrocarcins A–P, AC6H, arisostatins A and B which were isolated from *Micromonospora* bacteria [4,5,11,12,13,14]. Various studies reported that tetrocarcin A and its analogues had the antibiotic activity against several Gram-positive bacteria as well as anticancer activity [6,15,16]. Therefore, our objective is to discover new tetronolides with antibacterial activity, provide more information for the structure-activity relationship, as well as the possibility to improve their potential applications.

**Figure 1:**
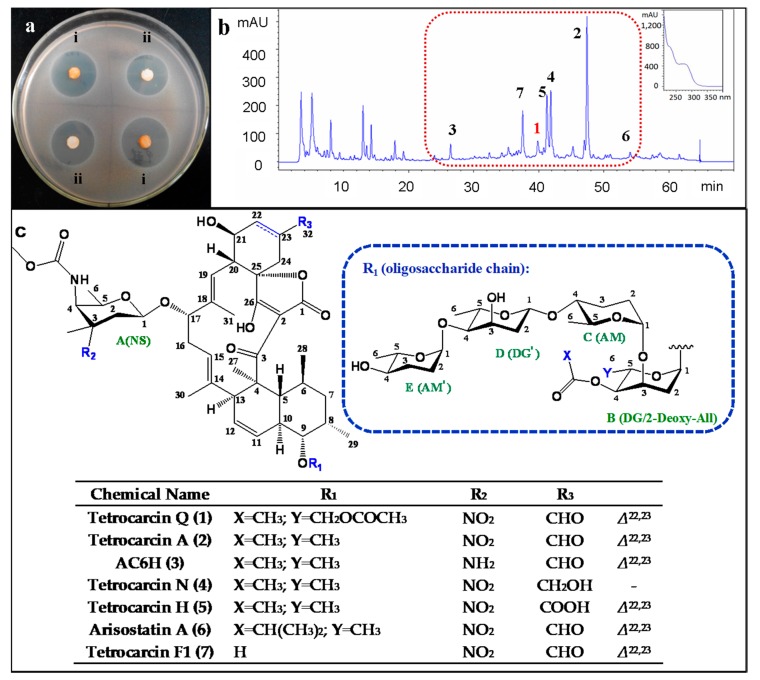
The bioassay and the HPLC fingerprint of the organic extracts, and the structures of the isolated compounds. (**a**) The antibacterial activity of the organic extracts (i: the ethyl acetate extract; ii: the methanol extract) against *B. subitlis* ATCC 63501 using paper disk method (5 mg/piece); (**b**) The HPLC fingerprint of the ethyl acetate extract, and the peaks of the tetrocarcins were marked in red box based on their UV spectra. Note: the peak numbers represent the structure numbers; (**c**) The chemical structures of **1**–**7** from *M. carbonacea* LS276. Note: the sugar types marked in green color include NS (tetronitrose), DG (digitoxose), 2-Deoxy-All (2-deoxy-allose), and AM (amicetose).

The ethyl acetate extract of the fermentation broth from the marine-derived *M. carbonacea* LS276 [17] showed antibacterial activity (Figure 1a). Bioassay-guided fractionation of the extract and further purification allowed for the isolation of seven spirotetronate glycosides (**1**–**7**). Among them, tetrocarcin Q (**1**) has a unique oligosaccharide chain at the C-9 position, which is different from other known spirotetronate glycosides. A major constituent, tetrocarcin A (**2**), was found to be the main active component of this strain (Figure 1b,c). Herein, we report the isolation, structure elucidation, and biological activities of these compounds.

## 2. Results and Discussion

### 2.1. Structure Elucidation of Compounds

**Figure 2:**
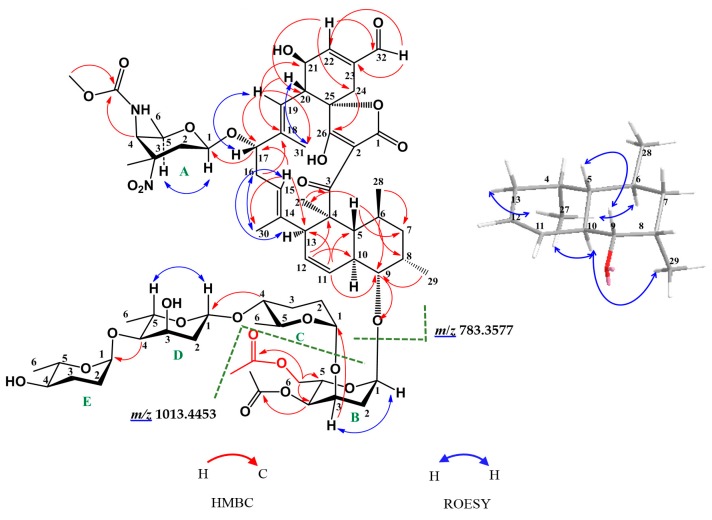
Key HMBC (red arrows) and ROESY (blue arrows) correlations of tetrocarcin Q (**1**).

Compound **1** was obtained as a white powder. Its molecular formula C_69_H_98_N_2_O_26_ was determined by the (+)-HR-ESI-MS peak at *m*/*z* 1393.6260 [M + Na]^+^, indicating 22 degrees of unsaturation. The ^1^H NMR spectrum of **1** (Table 1, Supplementary Figure S2) displayed one aldehydic proton at δ_H_ 9.58 (s, H-32), five olenic protons (δ_H_: 5.74 (d, *J* = 10.2 Hz, H-11), 5.42 (m, H-12), 5.16 (m, H-15), 5.21 (d, *J* = 10.2 Hz, H-19), 6.92 (s, H-22)), five glycosyl anomeric protons (δ_H_: 4.44 (dd, *J* = 9.6, 1.8 Hz, H-A-1), 4.92 (d, *J* = 4.8 Hz, H-B-1), 4.88 (brd, *J* = 3.0 Hz, H-C-1), 4.90 (dd, *J* = 9.6, 1.8 Hz, H-D-1), 4.91 (brs, H-E-1)), one methoxy group at δ_H_ 3.71 (s, H-A4-NHCOOCH_3_), six methyl singlets (δ_H_: 2.08 (H-B4-OCOCH_3_), 2.07 (H-B6-OCOCH_3_), 1.63 (H-27), 1.60 (H-A3-CH_3_), 1.53 (H-31), 1.34 (H-30)), and six methyl doublets (δ_H_: 1.32(d, *J* = 6.0 Hz, H-D-6), 1.23 (d, *J* = 6.0 Hz, H-E-6), 1.16 (d, *J* = 6.6 Hz, H-C-6), 1.15 (d, *J* = 6.6 Hz, H-A-6), 1.09 (d, *J* = 7.2 Hz, H-29), 0.64 (d, *J* = 6.0 Hz, H-28)). The ^13^C NMR (Table 1, Supplementary Figure S3) and Heteronuclear Single Quantum Coherence (HSQC) (Supplementary Figure S4) spectra revealed 69 carbon signals, including seven carbonyls or keto-enolic carbons (δ_C_: 206.4, 201.5, 192.6, 170.9, 170.2, 166.7, 157.4), nine olefinic carbons (δ_C_: 149.6, 141.6, 136.5, 136.1, 126.2, 126.1, 123.1, 118.3, 100.9), five sugar anomeric carbons (δ_C_: 99.5, 98.9, 96.5, 92.7, 92.0), one methoxyl (δ_C_: 53.0), 12 methyls (δ_C_: 25.4, 22.1, 21.0, 20.9, 19.0, 18.2, 17.8, 17.1, 16.3, 15.2, 14.5, 14.1). Comprehensive analysis of the ^1^H-^1^H Homonuclear chemical shift Correlation Spectroscopy (COSY) (Supplementary Figure S6), HSQC (Supplementary Figure S4) and Heteronuclear Multiple Bond Correlation (HMBC) (Supplementary Figure S5) spectra of **1**, indicated the presence of a spiroteronate skeleton, a tetronitrose (NS), and a tetrasaccharide, which is similar to tetrocarcin A (**2**). The difference between **1** and tetrocarcin A (**2**) is that the 6-methyl group (δ_H_ 1.13 (3H, d, *J* = 6.6 Hz); δ_C_ 17.6) of one digitoxose unit (sugar B) in tetrocarcin A (**2**) is replaced by a 6-oxymethylene (δ_H_ 4.32 (1H, dd, *J* = 12.0, 5.4 Hz) and 4.12 (1H, dd, *J* = 12.0, 1.8 Hz); δ_C_ 63.4) and an acetyl group (δ_H_ 2.07 (3H, s); δ_C_ 21.0, 170.9) (Table 1, Supplementary Figure S10, Supplementary Table 1). The HMBC correlations of 6-oxymethylene protons (δ_H_ 4.32 and 4.12) to the ester carbonyl (δ_C_ 170.9, B6-OCOCH_3_) and two oxygenated-carbons (δ_C_ 69.5, B-4 and δ_C_ 64.5, B-5) confirmed that the first sugar (sugar B) is 4,6-*O*-diacetyl-2-deoxysugar, which was supported by the (+)-HR-ESI-MS fragments (*m*/*z* 1013.4453 and 783.3577) corresponding to the ion of a subunit consisting of the spiroteronate skeleton with NS (sugar A) and 4,6-*O*-diacetyl-sugar (sugar B), and a subunit of the spiroteronate skeleton with NS (Figure 2, Supplementary Figure S1). On the basis of the above information, all protons and carbon resonances were assigned and the planar structure of **1** was established.

The relative configuration of **1** was the same as those of the previously reported tetrocarcins, based on the similarity of their NMR spectral data of the aglycone, which was further supported by key coupling constants in ^1^H NMR spectrum and the correlations observed in the Rotating Frame Overhauser Effect Spectroscopy (ROESY) experiments (Figure 2, Supplementary Figure S7). The *Z*-configuration of the *Δ*^11,12^ double bond was assigned on the basis of the coupling constant *J*_H-11/H-12_ = 10.2 Hz. The ROESY correlations of H-13/H-15, H-16/H-30, H-17/H-19, and H-31/H-20 indicated that the *E*-configurations of the both double bonds *Δ*^14,15^ and *Δ*^18,19^. In addition, the ROESY correlations of H-13/H-27, H-27/H-10, H-27/H-6, and H-29/H-10 revealed that these protons were on the same side of the decalin ring, whereas the ROESY correlations of H-9/H-5 indicated that they were on the other side of the ring. The absolute configurations of the stereogenic carbons in the aglycone of **1** were the same as those of tetrocarcin A (**2**), since they displayed similar circular dichroism (CD) curves, which showed a negative Cotton effect at 224 nm and a positive Cotton effect at 264 and 300 nm [4] (Supplementary Figure S8). The relative configurations of sugars A-E were determined as β, α, α, β, and α-orientations by the coupling constants of the anomeric protons (δ_H_: 4.44 (dd, *J* = 9.6, 1.8 Hz, H-A-1), 4.92 (d, *J* = 4.8 Hz, H-B-1), 4.88 (brd, *J* = 3.0 Hz, H-C-1), 4.90 (dd, *J* = 9.6, 1.8 Hz, H-D-1), 4.91 (brs, H-E-1)), which was confirmed by the ROESY correlations (H-A-1/H-A-5, H-B-1/H-B-3, H-D-1/H-D-5).

Compounds **2**–**7** were also obtained as white amorphous powders, and they were identified as tetrocarcin A (**2**), AC6H (**3**), tetrocarcin N (**4**), tetrocarcin H (**5**), arisostatin A (**6**), and tetrocarcin F1 (**7**) by comparison of their spectral data (MS, ^1^H, ^13^C NMR, specific rotation) with those reported in the literature [4,5,12,13,14].

### 2.2. Biological Assays

All of the isolated compounds were evaluated for their antibacterial activity against *Bacillus subitlis* ATCC 63501, *Staphylococcus aureus* ATCC 29213, *Staphylococcus epidermidis* ATCC 12228, *Enterococcus faecalis* ATCC 29212, *Pseudomonas aeruginosa* ATCC 27853, and *Escherichia coli* ATCC 25922. Except for **7**, the other six compounds exhibited antibacterial activity against *B. subtilis* with minimum inhibitory concentration (MIC) from <0.048 μM to 50 μM, with **2** and **6** showing strong antibacterial activity (Table 2). The MIC values of **2**–**7** were found to be similar to those previously reported [4,9,11,14,18].

Compound **7** exhibited no activity, indicating that the oligosaccharide chain is required for the antibacterial activity. Compound **3** was at least 10-fold less active than **2**, suggesting that the NO_2_-sugar is also important for the antibacterial activity. Compounds **4** and **5** were less active than **2**, **3** and **6**, inferring that the aldehyde group at C-23 is also essential for the activity. These results are all in accordance with the previously reported structure-activity relationship [1,4,9,11,14].

Compound **1** displayed a moderate antibacterial activity with MIC value of 12.5 μM, which was less active than **2**, implying that 6-CH_3_ of sugar B in the oligosaccharide chain at C-9 plays a key role in the antibacterial activity (Table 2).

Compounds **1**–**7** were evaluated by MTT method for their in vitro antitumor activity against five human cancer cell lines including: human non-small cell lung cancer cell (A549), human gastric cancer cell (BGC823), human colonic carcinoma cell (HCT116), human hepatoma cell (HepG2), human glioblastoma multiform cell (U87MG). In addition to moderate activity against the other four cell lines with the IC_50_ values ranging from 5.33 μM to 19.7 μM, **2** and **6** exhibited the most potent antitumor activity against U87MG cell line with IC_50_ values of 0.50 μM and 2.42 μM, respectively (Table 3). The other compounds were considered to be weakly active or inactive (IC_50_ > 10 μM).

The structure-activity relationship of the seven compounds on the human tumor cell lines A549, BGC823, HCT116, HepG2 and U87G was very similar to that obtained from antibacterial assay against *B. subitlis* (Table 2 and Table 3). The most active compounds were **2** and **6**, which was in agreement with the previous studies [9]. The activities of **3**, **4** and **5** were decreased, suggesting the modification of the tetronolide skeleton have influence on the in vitro antitumor activity in some extent. The lack of activity of **7** implies that the sugar moiety at C-9 position could play an important role in the antitumor activity, which was also in agreement with the previous structure-activity relationship study [15]. Compound **1** showed no or weak in vitro antitumor activity, suggesting that the deoxy sugar analogue may also influence the antitumor activity.

## 3. Materials and Methods

### 3.1. General

Optical rotations were measured on a JASCO P-2000 digital polarimeter (JASCO Corporation, Tokyo, Japan). Circular dichroism (CD) spectrum was recorded using a JASCO J-815 CD spectro polarimeters (JASCO Corporation, Tokyo, Japan). ^1^H and ^13^C NMR, and 2D NMR spectra were obtained at 600 and 150 MHz, using a Bruker AVANCE 600-III spectrometer (Bruker Biospin Group, Karlsruhe, Germany) in chloroform-*d* with TMS as an internal reference. HR-ESI-MS data were measured using an Agilent 1100 LC/MSD Trap SL LC/MS/MS spectrometer (Agilent Technologies, Santa Clara, CA, USA). Semipreparative HPLC was performed by an Agilent 1200 HPLC system (Agilent Technologies, Santa Clara, CA, USA) using a Shiseido Capcell Pak C18 column (5 μm, 10 × 250 mm). Column chromatography was performed with RP-18 (40–60 μm, GE healthcare, Fairfield, CT, USA) and Sephadex LH-20 (18–110 μm, GE healthcare, Fairfield, CT, USA).

### 3.2. Bacterial Material and Fermentation

The strain LS276 was isolated from a sponge *Gelliodes carnosa* collected from Ling shui Bay, Hainan Province of China near Xincun Harbor (18°24′5.49″ N, 109°59′37.76″ E), in August 2007 [17]. It was identified as *M. carbonacea* based on the morphology and 16S rRNA gene sequence analysis. The DNA sequence was deposited in GenBank (Accession No. FJ937935.1). The strain LS276 was first cultivated on agar plates (medium: starch 40.0 g; glucose 0.5 g; peptone 5.0 g; soybean powder 5.0 g; CaCO_3_ 1.0 g; K_2_HPO_4_ 0.5 g; MgSO_4_ 0.5 g; agar 10.0 g; distilled water 1 L; pH 7.0–7.2) at 28 °C for five days. Then, the mycelia were inoculated into 500-mL Erlenmeyer flasks, each containing 100 mL of liquid medium (composed of starch 40.0 g; glucose 0.5 g; peptone 5.0 g; soybean powder 5.0 g; CaCO_3_ 1.0 g; K_2_HPO_4_ 0.5 g; MgSO_4_ 0.5 g; distilled water 1 L; pH 7.0–7.2). The flasks were incubated at 28 °C on a rotary shaker (200 rpm) for three days. Seed culture (10 mL) was transferred into two hundred 500-mL Erlenmeyer flasks each containing 100 mL of fermentation medium (composed of 10.0 g of starch; 20.0 g of glucose; 5.0 g of soybean powder; 1.0 g of KNO_3_; 0.5 g of NaCl; 0.5 g of K_2_HPO_4_; 0.01 g of MgSO_4_ in 1 L of distilled H_2_O) and incubated at 28 °C on a rotary shaker (200 rpm) for nine days.

### 3.3. Extraction and Isolation

The culture broth (20 L) was repeatedly extracted with ethyl acetate (*v*/*v* 1:3, three times) by ultrasound, and the organic solvent was evaporated to dryness under a vacuum to afford the crude extract (4.0 g). The crude extract was first subjected to Sephadex LH-20 chromatography (3 × 60 cm, 100 g) using CH_3_OH (each 20 mL) as eluent and afforded six primary Fractions (Frs) 1–6. Fr.3 (2.0 g) was separated via semipreparative HPLC using 65% CH_3_CN in H_2_O, flow rate 2 mL/min as eluent to give Fr.3.1 to Fr.3.6. Fr.3.1 was further purified by semipreparative HPLC with a linear gradient of CH_3_CN–0.02%CH_3_COOH/H_2_O (60% to 100%, 35 min) to provide compounds **1** (2.4 mg, *t*_R_ = 10.0 min), **4** (12.6 mg, *t*_R_ = 10.8 min) and **5** (9.4 mg, *t*_R_ = 10.4 min). Fr.3.2 was further purified by semipreparative HPLC with a linear gradient of CH_3_CN–0.02%CH_3_COOH/H_2_O (65% to 72%, 40 min) to yield compound **2** (46.7 mg, *t*_R_ = 16.7 min). Fr.3.5 was further purified by semipreparative HPLC with a linear gradient of CH_3_CN–0.02%CH_3_COOH/H_2_O (65% to 72%, 40 min) to yield compound **6** (2.6 mg, *t*_R_ = 18.9 min). Fr.5. (300 mg) was purified by semipreparative HPLC afforded compound **3** (3.6 mg, linear gradient of 20–70% CH_3_CN in H_2_O for 50 min, flow rate 2 mL/min, *t*_R_ = 28.2 min). Purification of the Fr.4. (100 mg) by semipreparative HPLC provided compound **7** (7.8 mg, linear gradient of 20–70% CH_3_CN in H_2_O for 50 min, flow rate 2 mL/min, *t*_R_ = 37.3 min).

Tetrocarcin Q (**1**). White amorphous powder; ${\left[\alpha \right]}_{D}^{20}$ −80.9 (*c* 0.05, MeOH); UV (MeOH) λ_max_ (log ε) 203 (4.13), 242 (3.02), and 274 (4.07) nm; CD (*c* 0.5 (*w*/*v*)%, MeOH) 224 (−22.6), 264 (6.00), 343 (−0.85) nm; ^1^H NMR (CDCl_3_, 600 MHz) and ^13^C NMR (CDCl_3_, 150 MHz) data, Table 1; (+)-HR-ESI-MS *m*/*z* 1393.6260 [M + Na]^+^ (calcd. for C_69_H_98_N_2_O_26_Na, 1393.6270).

### 3.4. Biological Assays

Antibacterial and in vitro antitumor assays were performed for the isolated compounds with the purity of >90% by HPLC.

#### 3.4.1. Antibacterial Activity

The tested bacteria used in this study were as follows: *B. subitlis* ATCC 63501, *S. aureus* ATCC 29213, *S. epidermidis* ATCC 12228, *E. faecalis* ATCC 29212, *P. aeruginosa* ATCC 27853, and *E. coli* ATCC 25922, MIC values against the six bacterial strains were measured by using the 96-well plate-based assay [19]. The bacterial strains cultured in respective medium were collected at OD_600_ of 0.3–0.5, then further diluted to OD_600_ of 5 × 10^−4^. Aliquots of this suspension (100 μL) were placed into a 96-well plate. The tested compounds were added into the bacteria suspensions to give the desired concentration. The wells containing the same number of cells but no compounds were set as control groups. The positive control was ampicillin. The cultures were then added respective solutions and further incubated at 37 °C for 18 h. The plate was then read using a microplate reader at 600 nm. Each concentration had triplicate values, and the whole experiment was carried out at three times and the MIC value was determined by taking the average of triplicate OD_600_ values for each concentration and plotting it against concentration. The MIC value was determined, as the point in the curve where the OD_600_ is similar to that of control without bacteria.

#### 3.4.2. Antitumor Activity

The in vitro antitumor activity (represented by IC_50_ values) of the isolated compounds against five tumor cell lines, including A549, BGC823, HCT116, HepG2 and U87MG, was determined by MTT method as reported [20,21], and the dose-response curves were fitted with Sigma plot.

## 4. Conclusions

In summary, seven spirotetronate glycosides were isolated and characterized from the marine-derived *M. carbonacea* LS276. Among them, tetrocarcin Q (**1**) is a new compound. It is worth mentioning that the sugar B of compound **1** is 6-*O*-acetylated, while the other sugars in the previous spirotetronate glycosides are 6-deoxy sugars (DG and AM), which expanded the structural variability of such spirotetronate glycosides. We propose that the glycosyltransferase, especially TcaT3 [2], might recognize other sugar donors, just having a preference for digitoxose to biosynthesize its “natural and suitable” products. Another possibility is that the glycosyltransferase had accidentally evolved, which could identify other sugar donors. Efforts are underway to verify our inference through the in vitro enzymatic reaction. Thus, this study provides a new idea for the future biosynthesis of the novel and potential spirotetronate glycosides.

## Figures and Tables

**Table 1:** The ^1^H (600 MHz) and ^13^C NMR (150 MHz) data of tetrocarcin Q (**1**) in CDCl_3_. Underline: the NMR data (3.71, 53.0) referred to the group CH_3_ in underline, while 157.4 referred to CO in underline.

No.	*δ*_H_ Mult. (*J* in Hz)	*δ*_C_	No.	*δ*_H_ Mult. *(J* in Hz)	*δ*_C_
**Spiroteronate Skeleton**					
1	-	166.7	17	4.28, brs	78.0
2	-	100.9	18	-	141.6
3	-	206.4	19	5.21, d (10.2)	118.3
4	-	51.3	20	3.06, t (9.6)	45.0
5	2.07, m	43.4	21	4.85, m	69.2
6	1.37, m	31.3	22	6.92, s	149.6
7	1.46, m; 1.60, m	41.6	23	-	136.5
8	2.20, m	34.5	24	2.56, m; 2.83, dt (2.5,18.9)	29.8
9	3.49, dd (5.1, 10.5)	84.8	25	-	84.1
10	2.10, t (9.8)	38.5	26	-	201.5
11	5.74, d (10.2)	126.1	27	1.63, s	15.2
12	5.42, m	126.2	28	0.64, d (6.0)	22.1
13	3.28, m	54.3	29	1.09, d (7.2)	14.1
14	-	136.1	30	1.34, s	14.5
15	5.16, m	123.1	31	1.53, s	16.2
16	2.28, m; 1.59, m	30.8	32	9.58, s	192.6
**Sugars**					
A-1	4.44, dd (9.6, 1.8)	96.5	C-1	4.88, brd (3.0)	92.7
A-2	2.72, brd (9.6); 1.64, m	36.1	C-2	1.88, m; 1.75, m	29.6
A-3	-	91.6	C-3	2.03, m; 1.97, m	26.4
A-4	4.36, dd (10.2, 2.4)	53.8	C-4	3.21, td (9.6, 4.8)	81.3
A-4-NH	5.07, d (10.2)		C-5	3.70, m	68.1
A-5	3.48, m	69.4	C-6	1.16, d (6.6)	18.2
A-6	1.15, d (6.6)	17.1	D-1	4.90, dd (9.6, 1.8)	99.5
A3-CH_3_	1.60, s	25.4	D-2	2.15, dt (14.4, 1.8);1.67, m	37.1
A4-NHCOOCH_3_	3.71, s	53.0	D-3	4.25, m	64.0
A4-NHCOOCH_3_	-	157.4	D-4	3.46, dd (9.6, 3.0)	75.3
B-1	4.92, d (4.8)	98.9	D-5	3.85, dq (9.6, 6.0)	67.9
B-2	2.24, dd (14.4, 3.0); 1.79, m	31.2	D-6	1.32, d (6.0)	19.0
B-3	4.23, m	66.5	E-1	4.91, brs	92.0
B-4	4.83, dd (10.5, 3.0)	69.5	E-2	1.83, 2H, m	29.8
B-5	4.50, m	64.6	E-3	1.90, m; 1.74, m	27.5
B-6	4.32, dd(12.0, 5.4);	63.5	E-4	3.30, td (9.6, 4.8)	71.8
	4.12, dd (12.0, 1.8)				
B4-OCOCH_3_	2.08, s	20.9	E-5	3.63, dq (9.6, 6.0)	70.4
B4-OCOCH_3_	-	170.2	E-6	1.23, d (6.0)	17.8
B6-OCOCH_3_	2.07, s	21.0	-	-	-
B6-OCOCH_3_	-	170.9	-	-	-

**Table 2:** Minimum inhibitory concentrations (MICs) (μM) for *B. subitlis* ATCC 63501 of compounds **1**–**7**.

Compounds	MICs (μM)							
	1	2	3	4	5	6	7	Ampicillin
*B. subitlis* ATCC 63501	12.5	<0.048	0.5	1.562	50	0.048	>400	3.125

**Table 3:** In vitro antitumor activity (IC_50_, μM*)* of compounds **1**–**7**.

Compounds	IC_50_ (μM)				
	A549	BGC823	HCT116	HepG2	U87 MG
**1**	>50.0	28.3	32.4	49.3	13.3
**2**	5.71	7.45	5.97	18.2	0.50
**3**	19.2	25.4	28.2	>50.0	11.0
**4**	27.1	27.4	27.3	>50.0	21.3
**5**	>50.0	>50.0	>50.0	>50.0	44.7
**6**	5.33	19.7	6.53	18.8	2.42
**7**	>50.0	>50.0	>50.0	>50.0	>50.0
**paclitaxel ^a^**	0.001	0.01	0.01	0.07	-
**gefitinib ^b^**	-	-	-	-	8.30

^a^ Positive control used in A549, BGC823, HCT116 and HepG2 cell lines; ^b^ Positive control used in U87MG cell line.

## Supplementary Materials

The following are available online at http://www.mdpi.com/1660-3397/16/2/74/s1, Figure S1: The (+)-HRESIMS spectrum of tetrocarcin Q (**1**); Figure S2: The ^1^H NMR spectrum of tetrocarcin Q (**1**); Figure S3: The ^13^C NMR spectrum of tetrocarcin Q (**1**); Figure S4: The HSQC spectrum of tetrocarcin Q (**1**); Figure S5: The HMBC spectrum of tetrocarcin Q (**1**); Figure S6: The ^1^H-^1^H COSY spectrum of tetrocarcin Q (**1**); Figure S7: The ROESY spectrum of tetrocarcin Q (**1**); Figure S8: The CD spectrum of tetrocarcin Q (**1**); Figure S9: The CD spectra of compounds **1**–**7**; Figure S10: The ^13^C NMR difference spectra of tetrocarcin Q (**1**) and tetrocarcin A (**2**); Table S1: The ^1^H and ^13^C NMR different data for tetrocarcin Q (**1**) and tetrocarcin A (**2**).
