# Supplementary material for: Tetrocarcin Q, a New Spirotetronate with a Unique Glycosyl Group from a Marine-Derived Actinomycete Micromonospora carbonacea LS276
Source: Mar Drugs. 2018 Feb 24;16(2):74. doi: 10.3390/md16020074 (PMC5852502; doi:10.3390/md16020074)
Supplement: Supplementary file 1 [file marinedrugs-16-00074-s001.pdf]

Supplementary Information

Figure S1. The (+)-HRESIMS spectrum of tetrocarcin Q (**1**)

Figure S2. The ^{1}H NMR spectrum of tetrocarcin Q (**1**)

Figure S3. The ^{13}C NMR spectrum of tetrocarcin Q (**1**)

Figure S4. The HSQC spectrum of tetrocarcin Q (**1**)

Figure S5. The HMBC spectrum of tetrocarcin Q (**1**)

Figure S6. The ^{1}H-^{1}H COSY spectrum of tetrocarcin Q (**1**)

Figure S7. The ROESY spectrum of tetrocarcin Q (**1**)

Figure S8. The CD spectrum of tetrocarcin Q (**1**)

Figure S9. The CD spectra of compounds **1 - 7**

Figure S10. The ^{13}C NMR difference spectra of tetrocarcin Q (**1**) and tetrocarcin A (**2**)

Table S1. The ^{1}H and ^{13}C NMR different data for tetrocarcin Q (**1**) and tetrocarcin A (**2**)

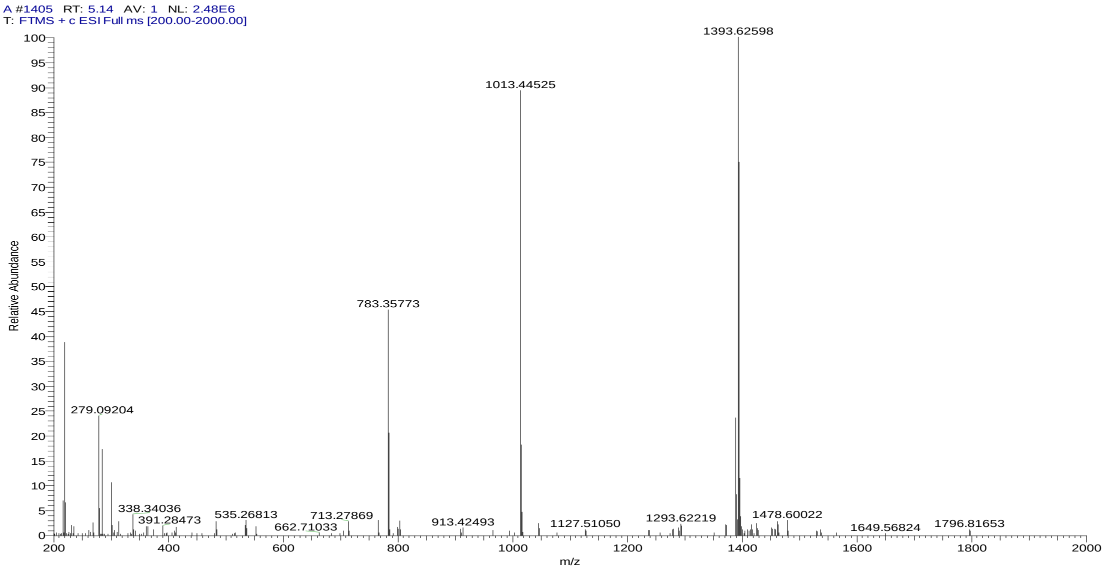

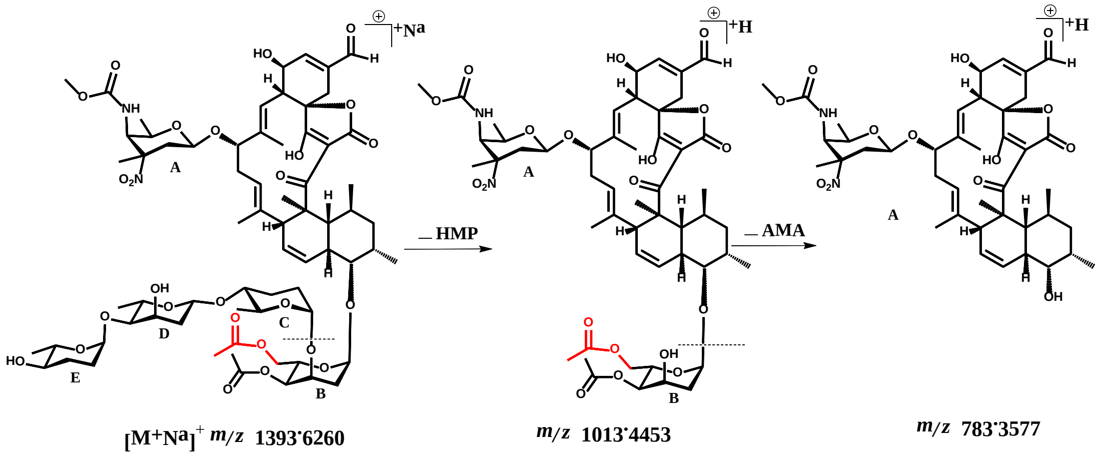

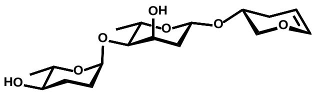

HMP=(2S,3R,6S)-6-(((2S,3R,4R,6R)-4-hydroxy-2-methyl-6-(((2S,3R)-2-methyl-3,4-dihydro-2H-pyran-3-yl)oxy)tetrahydro-2H-pyran-3-yl)oxy)-2-methyltetrahydro-2H-pyran-3-ol

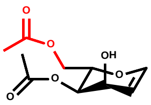

AMA=((2S,3R,4R)-3-acetoxy-4-hydroxy-3,4-dihydro-2H-pyran-2-yl)methyl acetate

Figure S1. The (+)-HRESIMS spectrum of tetrocarcin Q (**1**)

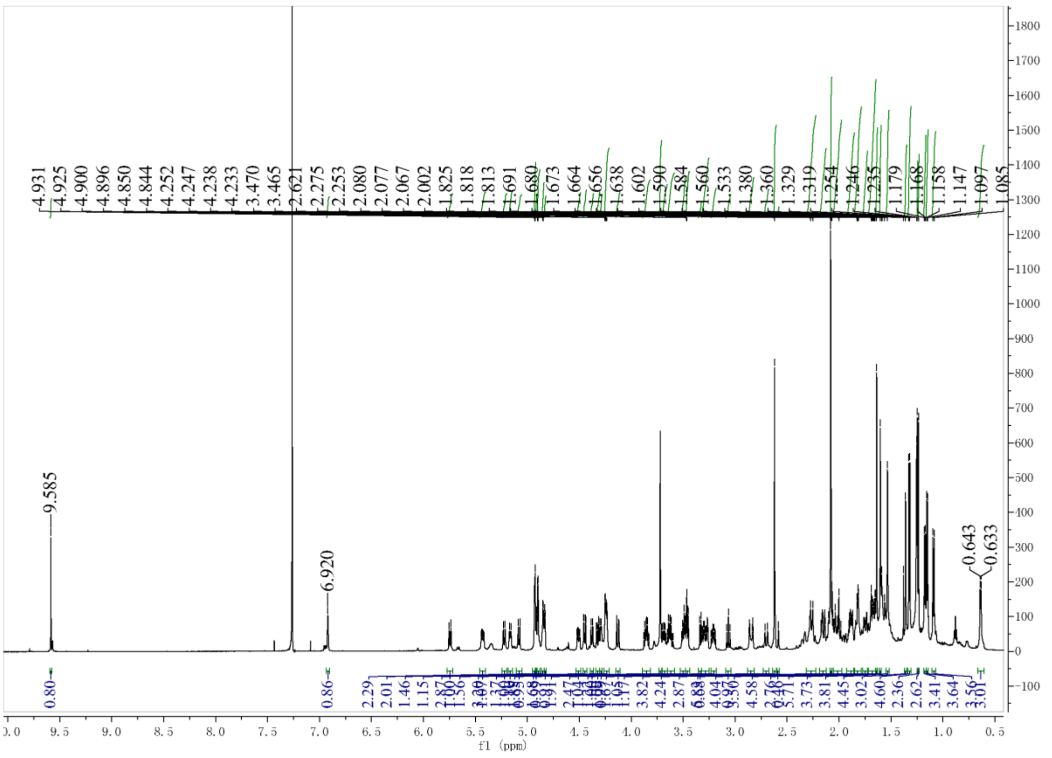

Figure S2. The ^{1}H NMR spectrum of tetrocarcin Q (**1**)

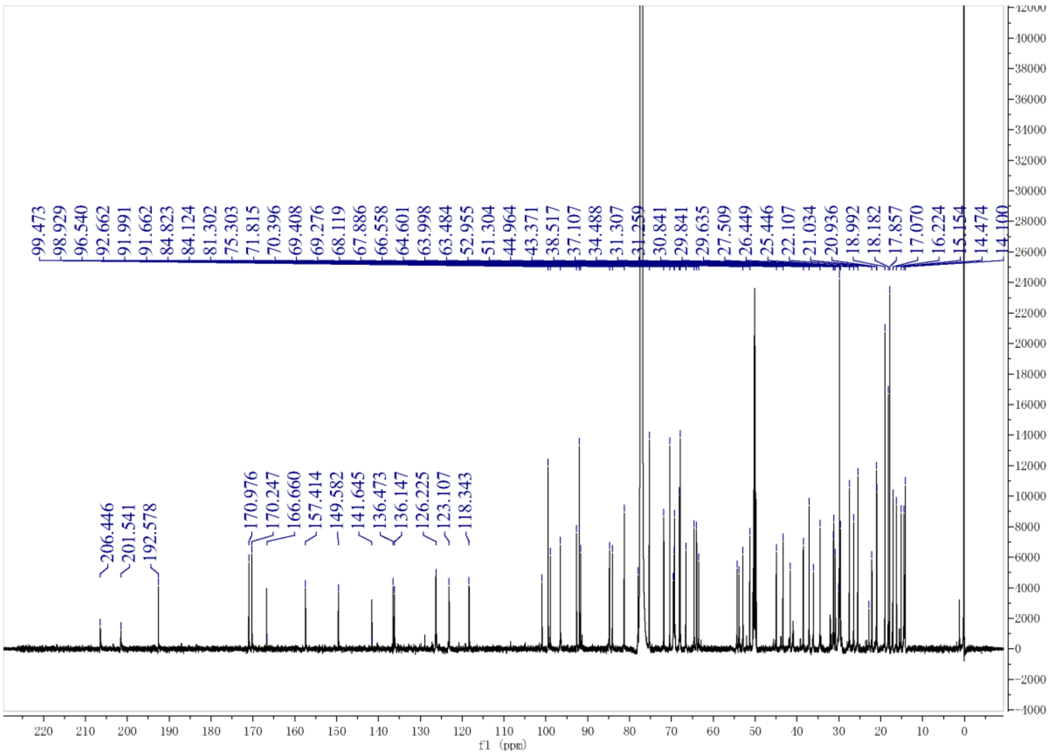

Figure S3. The ^{13}C NMR spectrum of tetrocarcin Q (**1**)

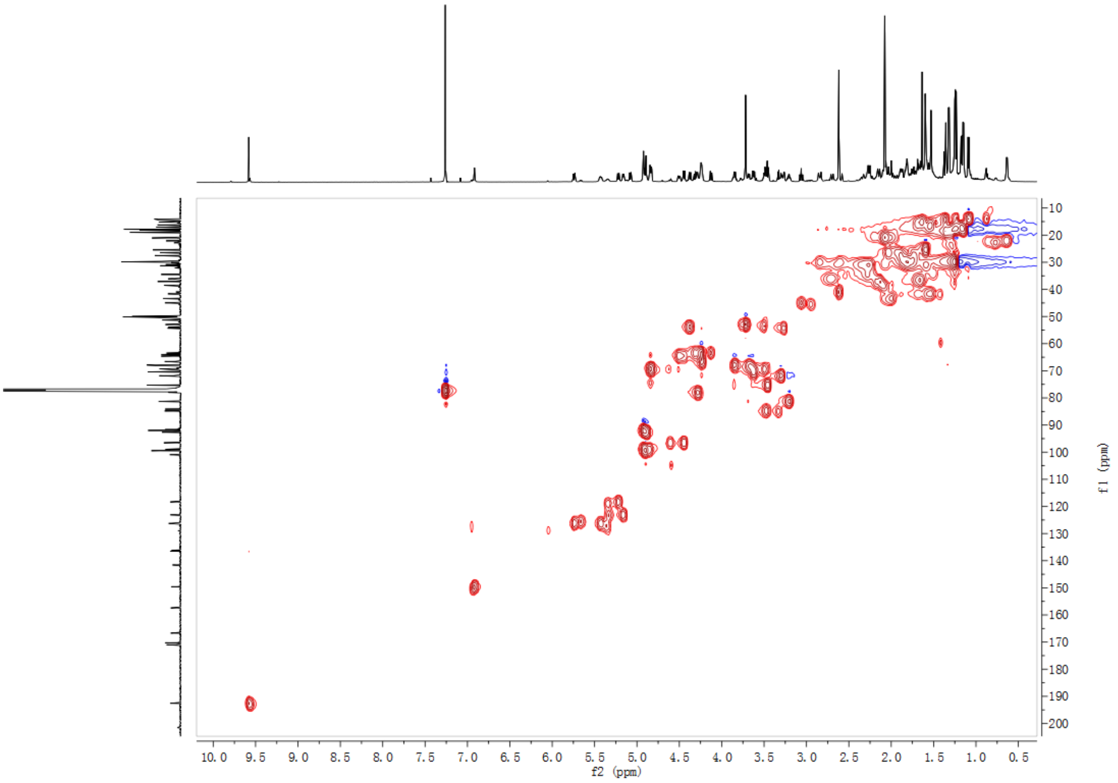

Figure S4. The HSQC spectrum of tetrocarcin Q (**1**)

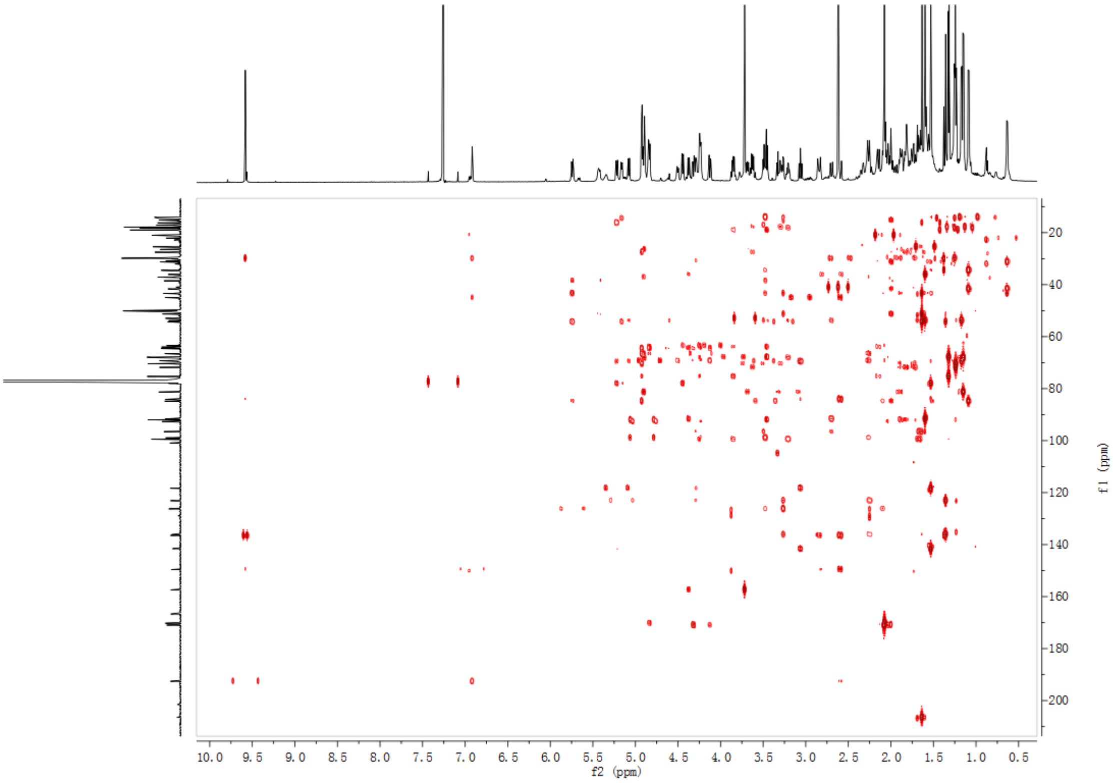

Figure S5. The HMBC spectrum of tetrocarcin Q (**1**)

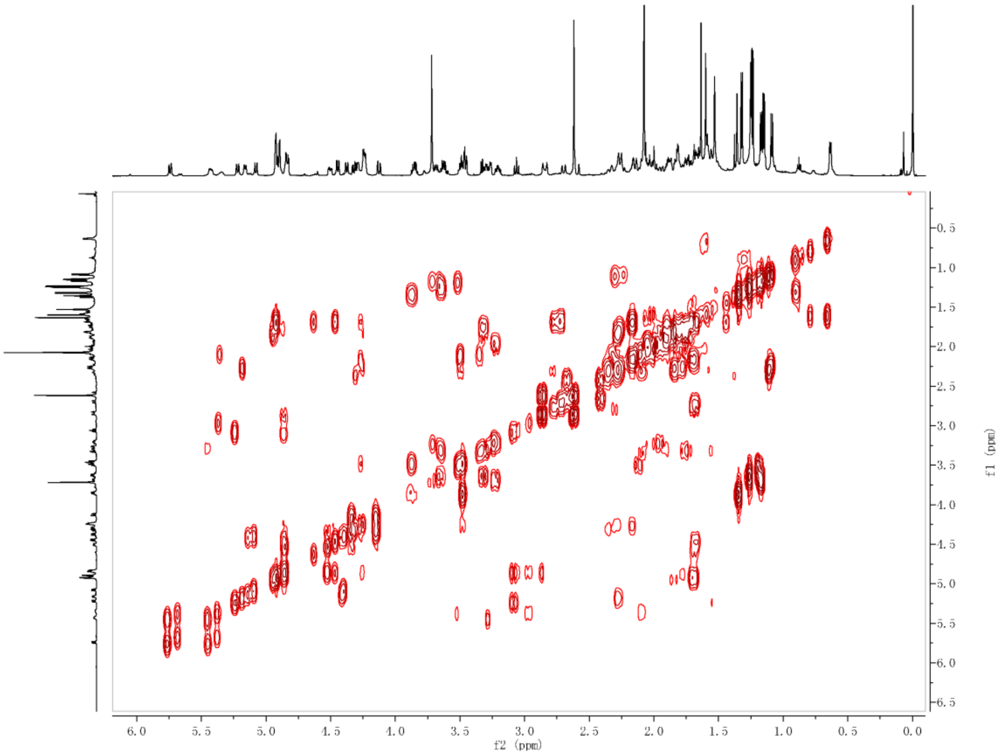

Figure S6. The ^{1}H-^{1}H COSY spectrum of tetrocarcin Q (**1**)

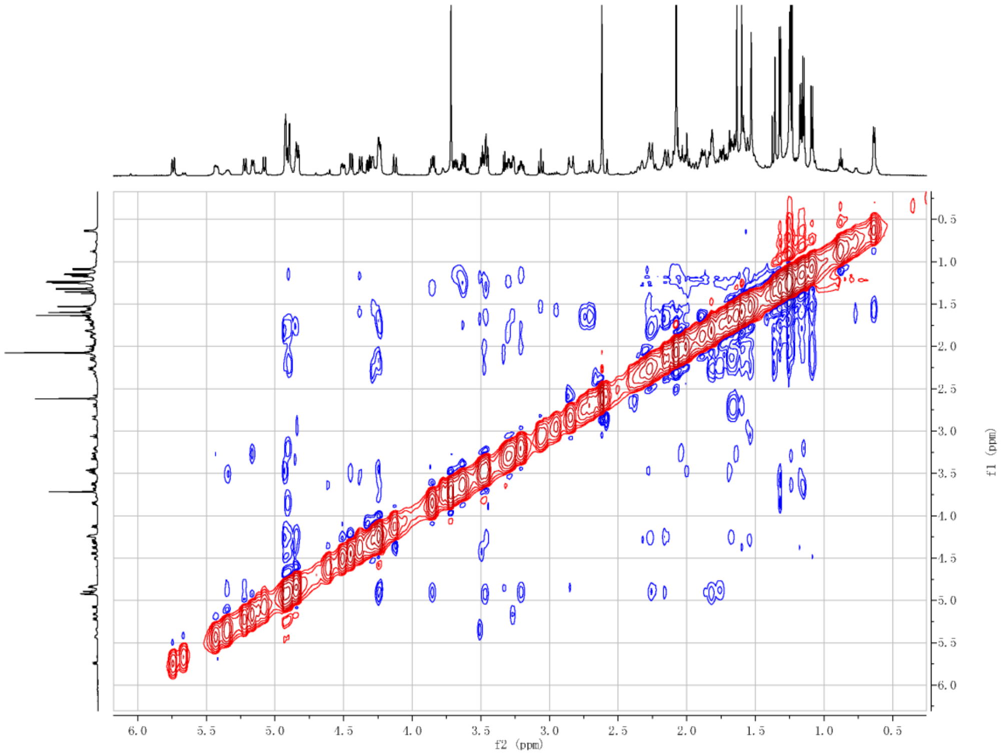

Figure S7. The ROESY spectrum of tetrocarcin Q (**1**)

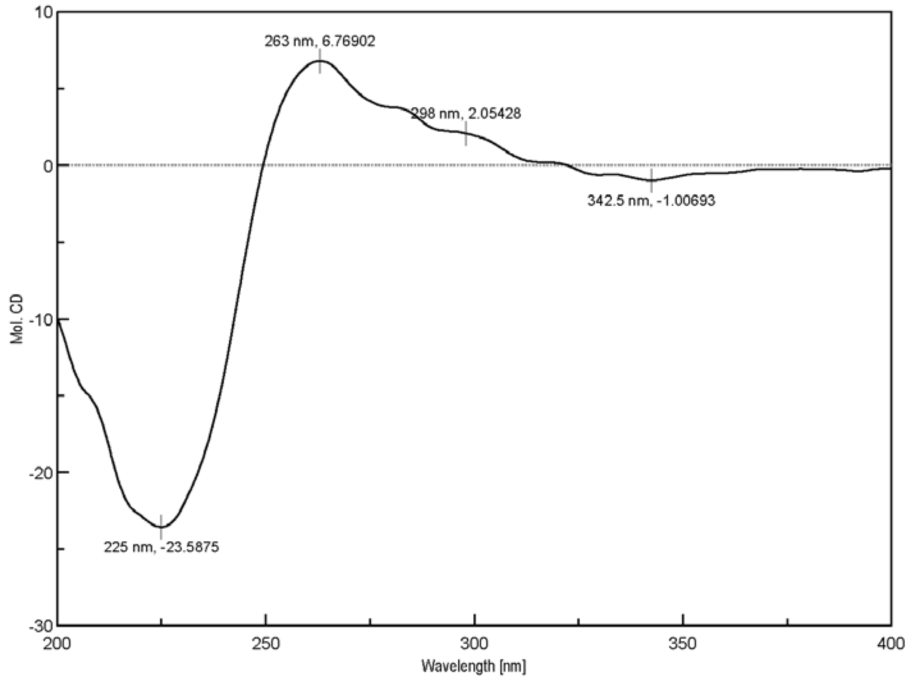

Figure S8. The CD spectrum of tetrocarcin Q (**1**)

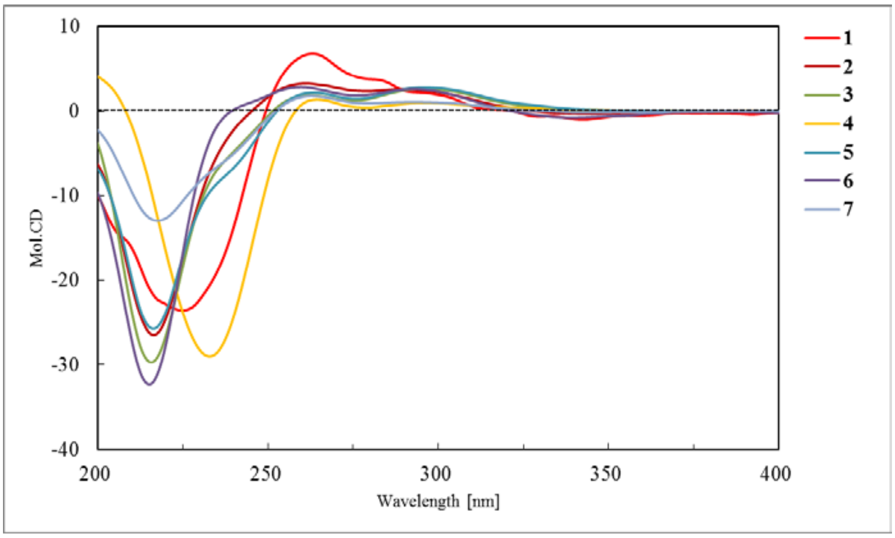

Figure S9. The CD spectra of compounds **1** - **7** (c 0.5(w/v)%, MeOH)

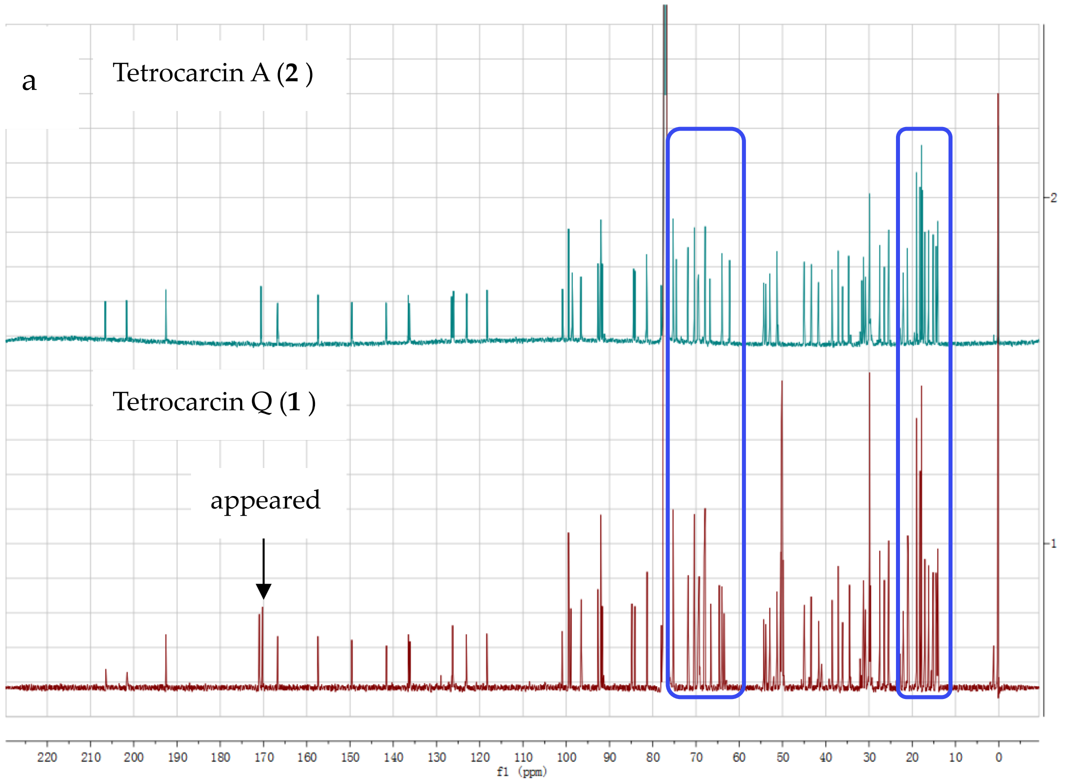
a
Tetrocarcin A (2)
Tetrocarcin Q (1)
appeared
220 210 200 190 180 170 160 150 140 130 120 110 100 90 80 70 60 50 40 30 20 10 0
f1 (ppm)

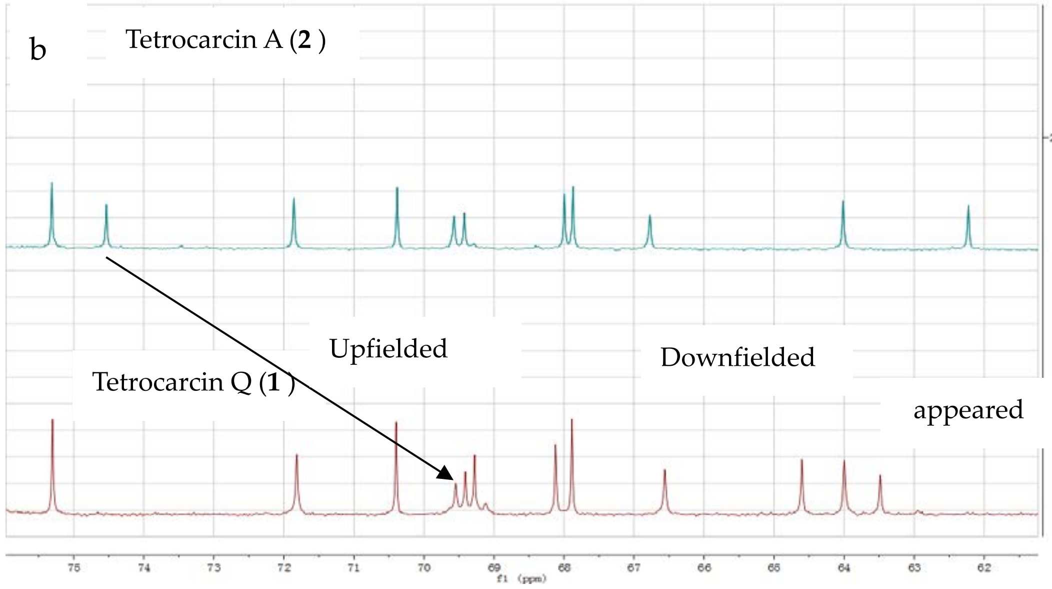
b
Tetrocarcin A (2)
Upfielded
Downfielded
Tetrocarcin Q (1)
appeared
75 74 73 72 71 70 69 68 67 66 65 64 63 62
f1 (ppm)

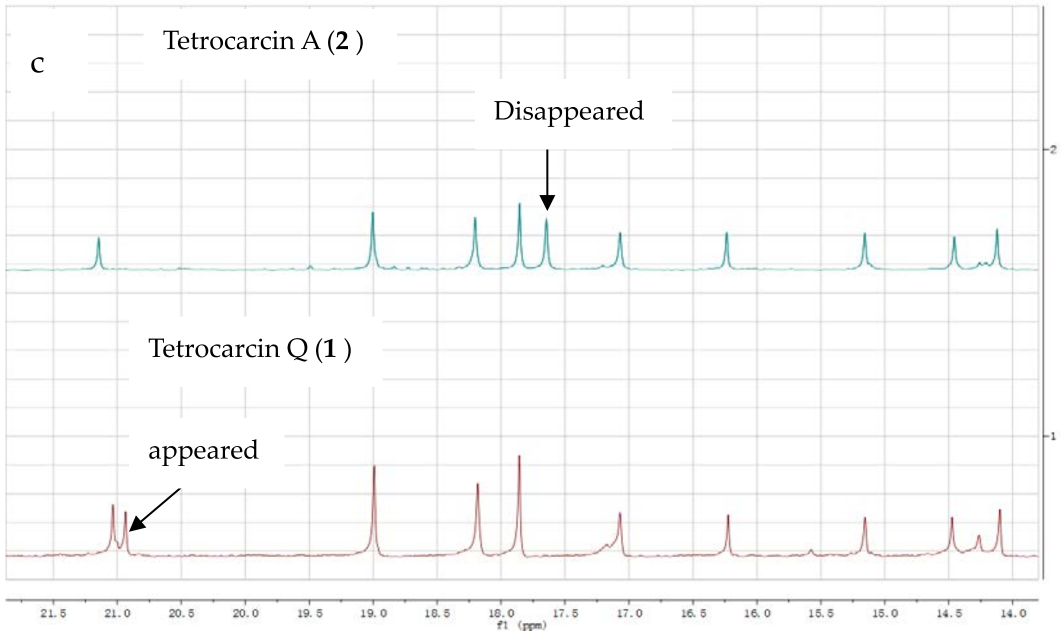

Figure S10. The ^{13}C NMR difference spectra of tetrocarcin Q (**1**) and tetrocarcin A (**2**). **a)** Full spectra; **b)** Partial spectra in 61.5 – 75.8 ppm; **c)** Partial spectra in 14.0 – 21.5 ppm.

Table S1. The ^{1}H and ^{13}C NMR different data for tetrocarcin Q (**1**) and tetrocarcin A (**2**).

	tetrocarcin Q (**1**)		tetrocarcin A (**2**)	
No.	δ_H mult. (*J* in Hz)	δ_C	δ_H mult. (*J* in Hz)	δ_C
B-1	4.90, d (4.8)	98.9	4.83, d (4.8)	98.6
B-2	2.24, dd (14.4, 3.0); 1.79, m	31.2	2.24, dd (14.4, 3.0); 1.79, m	31.3
B-3	4.23, m	66.5	3.85, m	66.6
B-4	4.83, dd (10.5, 3.0)	69.5	4.57, dd (9.6, 3.0)	74.5
B-5	4.50, m	64.6	4.36, m	62.2
B-6	4.32,dd(12.0,5.4); 4.12,dd (12.0,1.8)	63.5	1.13,d(6.6)	17.6
B4-OCO$\underline{CH_3}$	2.08, s	20.9	2.09, s	21.2
B4-O$\underline{CO}$$CH_3$		170.2		170.6
B6-OCO$\underline{CH_3}$	2.07, s	21.0		
B6-O$\underline{CO}$$CH_3$		170.9		
